# Implementing systems medicine within healthcare

**DOI:** 10.1186/s13073-015-0224-5

**Published:** 2015-09-29

**Authors:** Marc Kirschner, Angela Bauch, Alvar Agusti, Sebastian Hilke, Sibylle Merk, Christophe Pison, Jim Roldan, Bernard Seidenath, Michael Wilken, Emiel F. Wouters, Hans-Werner Mewes, Klaus Heumann, Dieter Maier

**Affiliations:** Forschungszentrum Jülich GmbH, Jülich, Germany; Biomax Informatics AG, Planegg, Germany; Hospital Clinic, Barcelona, Spain; BTA Bayerische Telemed Allianz, Ingolstadt, Germany; Reha-Zentrum Bad Griesbach, Bad Griesbach-Therme, Germany; Clinique Universitaire de Pneumologie, CHU de Grenoble, La Tronche, France; Linkcare Health Services, Barcelona, Spain; CSU-Bürgerbüro, Dachau, Germany; Deutsche Patientenliga Atemwegserkrankungen e.V, Oppenheim, Germany; CIRO+, Centre of Expertise for Chronic Organ Failure, Horn, The Netherlands; Helmholtz Zentrum München GmbH, Neuherberg, Germany

## Abstract

The cause of a complex disease cannot be pinpointed to a single origin; rather, a highly complex network of many factors that interact on different levels over time and space is disturbed. This complexity requires novel approaches to diagnosis, treatment, and prevention. To foster the necessary shift to a pro-active systems medicine, proof-of-concept studies are needed. Here, we highlight several systems approaches that have been shown to work within the field of respiratory medicine, and we propose the next steps for broader implementation.

## Complex disease management

Complex, non-communicable diseases, such as cardiovascular disease, cancers, diabetes, and chronic respiratory diseases (for example, chronic obstructive pulmonary disease (COPD)), account for more than 60 % of deaths worldwide [1]. Complex diseases result from multiple, dynamic interactions among various factors at different spatiotemporal levels, and the classical or reductionist approach of treating one disease in isolation and as if it has one origin is greatly challenged by a high degree of biocomplexity. Reliable diagnostic, therapy, and prevention strategies therefore need to integrate multiple factors that influence disease, whether of internal origin (for example, genetic predisposition, presence of biomarkers, or spatiotemporal dynamics) or external origin (for example, lifestyle, age, or fitness). This will allow a holistic understanding of health and disease and provide a view of healthcare that is focused on the patient.

To make systems medicine a reality, a cross-disciplinary effort is required. To cope with the enormous amount of information that modern life sciences are able to generate, clinicians need to integrate and interrelate data without becoming IT experts. They need multi-level intervention and prevention plans to monitor patient health states, supported by information systems that assist in making optimal decisions for diagnosis, treatment, and disease prevention. Such a development needs both healthcare organizations and reimbursement or insurance companies to reconsider the classical symptomatic-based intervention strategies, and to concentrate their attention on the holistic approaches that systems medicine is able to offer.

We provide examples of the opportunity offered by integrating knowledge from various healthcare aspects, ranging from clinical data to knowledge-based patient care, changes in lifestyle, clinical management and individual system-based decisions for treatment. We discuss how successful implementation of systems medicine needs not only suitable informatics tools but also a clear organizational infrastructure, both of which are dependent on a proper financial framework. We also propose a conceptual strategy for a systems-based healthcare framework that focuses on reference sites and a stepwise approach to implementation and scale-up.

## Proof of principle examples of systems medicine

Surveying the pilot projects for systems medicine within European healthcare, it appears that progress is being made in three areas: the use of reference sites that use a patient-centric approach, the use of disease models and technology to provide decision support software, and the use of knowledge-management systems. We provide more information on these areas below.

COPD affects about 10 % of the population and is a major, life-threatening disease. If the patient is to be placed at the center of the treatment plan, then a comprehensive understanding of the patient, their quality of life, their acute and chronic health problems, and their different co-morbidities is required. This is the approach taken by The Centre of Expertise for Chronic Organ Failure (CIRO). This is an expertise center for chronic disease care in The Netherlands and a pioneer in the field of systems medicine. At this site, an interdisciplinary team, together with the patient, generates an integrated care pathway built from multiple modules. The care pathway consists of a variety of basic modules covering different disease dimensions, such as dyspnea and exacerbation management, as well as coping and psychopathological aspects. Depending on the baseline assessment information, step-up modules may be added on top of this baseline management program. To enable such a patient-centric approach, CIRO needed to adapt workflows, processes, and decision-making procedures to enable a more holistic view of the patient’s health and disease status. The care management shifted from a fragmented disease-oriented approach to an integrated approach delivered by a multidisciplinary team of health care providers in partnership with the patients.

Most healthcare systems are divided into clinical specialties, which prevent a coordinated approach to therapy. The technology is now available, however, to shape an innovative healthcare framework that implements systems medicine concepts. Recently, several research projects funded by the European Commission (e.g., AirPROM, Synergy-COPD, and SysCLAD) have developed detailed computational models of whole-body systems that are able to lead to validated, personalized predictions for risk assessment and intervention strategies [2]. But beyond the understanding of disease models, such as those for COPD [2], technology needs to support individuals and care personnel in acting upon the information generated. Methods such as disease dashboards [3] and clinical decision support systems [4] provide technical solutions that can simplify and deliver complex information summaries and facilitate team-based approaches to care across multiple providers. Integration of these solutions into standard care-management processes is becoming the norm in some EU countries, such as the UK, although it is still lacking in others.

The need to avoid unnecessary treatment can hardly be overestimated. Operational excellence in terms of efficient and effective health care requires sophisticated knowledge management to improve patient care, clinical process management, and treatment strategies, and to offer transparency in outcomes. In the case of CIRO, a dedicated knowledge-management environment was applied for optimal integration of all relevant information, optimal patient stratification, and targeted therapy approaches. The knowledge-management environment offers the potential to analyze and integrate a wide variety of clinical and diagnostic variables and to relate program outcomes to patient characteristics and components of the management process. Initially focused on COPD, this system now serves as a blueprint to be applied in different disease areas and clinical settings.

The Clinic Hospital Barcelona followed a complementary approach to integrated care, which focused on the exchange of knowledge across tertiary hospitals and care personnel. A knowledge-sharing system enabled the transfer of care-plan and case-management information across the team. Key enabling factors were: the willingness or even pressure from paying institutions to include relevant outcome measures, such as patient adherence or reduced hospitalization, which are summarized in a multidimensional outcome index; changes to workflow and business processes that are fostered and implemented by the clinic management; and change management in terms of the integration of care personnel and patients into workflow planning and feedback.

## Proposed implementation strategy

Although the heterogeneity of European healthcare systems presents some obstacles, this diversity also provides enormous potential. It is important to support the exchange of experience in implementing new systems medicine infrastructures, to connect the initiatives and to harmonize the activities. In accordance with the European Innovation Partnership (www.ec.europa.eu) and the European Connected Health Alliance analysis on challenges and barriers to the introduction of eHealth (www.echalliance.com), we propose the following steps for the implementation of systems medicine in healthcare.

1. Establish stable local networks that continuously bring together the key stakeholders: patients, care managers, care personal, technology providers, entrepreneurs, policy makers, and regulators.
2. Implement reference sites to identify local barriers and challenges.
3. Exchange best-practice and experience between reference sites to identify patterns of barriers and possible solutions.
4. Provide required framework adaptations (legal, financial, regulatory, incentive, and educational systems) to enable scale-up and replication.

This conceptual framework should be complemented by strategies for implementing systems medicine approaches in healthcare as discussed below.

### Definition of success indicators

Indicators for a systems medicine approach to complex diseases need to be clearly defined, and both hospitals and insurance companies need to be able to make a proper assessment of their socio-economic benefits. A measurable positive impact on care and quality of life (based on quality-adjusted-life-years (QALY) and reduction in disease burden) could justify additional investments. We suggest building on the success of early prototypes based on patient-centric systems medicine that have provided tangible health and economic impacts as a promising way to move forward.

### Introduction of a measurable, multi-dimensional success index

In order to convince payers to support innovative systems approaches, a comprehensible, multidimensional success index [5] must be developed. The efficacy of complex interventions needs to be monitored together with the differential response to such interventions in patients with a multicomponent pathology such as COPD [6]. The preparedness of payers to spur such a success index will support, or even create, the necessary business process change necessary to further support a systems medicine approach. In addition, the efficiency indicators need to be clearly defined so that both hospitals and insurance companies are able to make a proper assessment of the socio-economic benefits of systems medicine approaches to complex diseases. At present, many healthcare systems still lack such an index and payers still hesitate to focus on true gains in treatment efficiency and patient quality of life. Instead, numbers of interventions and hospital days are too often the standard measures of efficiency.

### From reactive to pro-active medicine

**Fig. 1:**
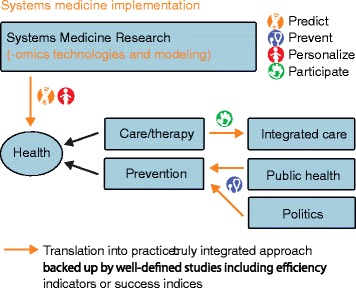
Implementing systems medicine in practice. Patient health is the driver for a future proactive P4 medicine that is predictive, preventive, personalized, and participatory [7]. This approach is supported by modern research, which includes both modern -omics technologies and mathematical or computational modeling, as an integral part of prediction and personalization. Integrated care concepts deliver a participatory aspect that needs support provided through prevention measures and proper back up from public health strategies and related political decisions

Lifelong, sustained patient health is the driver for the use of systems medicine in medical research and practice, and predictive, preventive, personalized, and participatory (P4 medicine [7]; Fig. 1) provides the most promising way forward for pro-active medicine. Environmental factors, such as diet, exercise, pollution, smoking, drug use, as well as intrinsic genetic disposition have a more pronounced impact on health than healthcare itself. Thus, a transition towards focusing on disease prevention or delaying disease onset will both contribute to individual health and reduce the burden of disease on society. Here, systems medicine has the potential to truly focus on the initial transition from wellness to disease and to identify possible prevention measures. This promises novel diagnostic and therapeutic strategies not only for preserving the wellness of each individual but also for promoting a return to a healthy state as soon as possible (Fig. 1).

## Conclusion

In some complex diseases, the practical feasibility of a systems medicine approach has already been proven successful [8–10]. In order to develop the full potential of systems medicine, a truly integrated approach involving all relevant stakeholders is needed that is backed up by well-defined studies, utilizing comprehensible efficiency indicators and success indices that demonstrate socio-economic benefits. Such a concept will have the greatest potential for systems-based medicine that can significantly reduce disease burden in the medium and long term.

## Abbreviations

CIRO: Centre of Expertise for Chronic Organ Failure
COPD: Chronic obstructive pulmonary disease
